# Is Active Synovitis of Metacarpophalangeal Joints a Neuropathic Condition in Rheumatoid Arthritis Patients? Results from an Ultrasound Study of Palmar Digital Nerves

**DOI:** 10.3390/jcm13061599

**Published:** 2024-03-11

**Authors:** Marco Di Carlo, Jacopo Di Battista, Edoardo Cipolletta, Tadashi Okano, Riccardo Chiorrini, Gianluca Smerilli, Francesca Bandinelli, Emilio Filippucci, Fausto Salaffi

**Affiliations:** 1 Rheumatology Unit, Università Politecnica delle Marche, Ospedale “Carlo Urbani”, 60035 Jesi, Italy; jacopeta@gmail.com (J.D.B.); edoardocipolletta@gmail.com (E.C.); okano.tadashi@omu.ac.jp (T.O.); chiorra96@gmail.com (R.C.); smerilli.gianluca@gmail.com (G.S.); emilio_filippucci@yahoo.it (E.F.); fausto.salaffi@gmail.com (F.S.); 2 Center for Senile Degenerative Disorders (CSDD), Osaka Metropolitan University Graduate School of Medicine, Osaka 545-8585, Japan; 3 Rheumatology Department, San Giovanni di Dio Hospital, USL Tuscany Center, 50143 Florence, Italy; francesca.bandi@gmail.com

**Keywords:** rheumatoid arthritis, palmar digital nerves, ultrasound, synovitis, disease activity

## Abstract

(1) **Background**: Rheumatoid arthritis (RA) is a chronic inflammatory joint disease, primarily characterized by pain. A significant proportion of patients report symptoms suggestive of neuropathic pain. The objectives of this study were to investigate the presence of an increased cross-sectional area (CSA) of the palmar digital nerves by ultrasound in patients with active synovitis of the metacarpophalangeal joints and to identify potential predictors of such an increase. (2) **Methods**: An ultrasound examination of the clinically most affected hand (from the second to the fifth metacarpophalangeal joint) was performed. The presence of synovitis was scored using a 0–3 semiquantitative method for each joint. The CSA of each pair of palmar digital nerves was measured. (3) **Results**: A significant correlation was found between the sum of the CSAs of the nerves and the Clinical Disease Activity Index (CDAI) (r = 0.387), as well as with the ultrasonographic grading of synovitis (r = 0.381) both at the patient and the joint level. These two variables, aimed at measuring disease activity, along with male gender, are the only predictors of the CSA of the palmar digital nerves. (4) **Conclusions**: Synovial inflammation of the metacarpophalangeal joints is, therefore, a condition that can influence the CSA of the palmar digital nerves and may partially explain neuropathic pain in patients with RA.

## 1. Introduction

Rheumatoid arthritis (RA) is a chronic inflammatory joint disease with high prevalence in the general population (1%). The diagnosis is currently based on the European Alliance of Associations for Rheumatology (EULAR)/American College of Rheumatology (ACR) 2010 criteria, integrating a clinical approach with laboratory and imaging techniques aimed at detecting synovitis. Early diagnosis is crucial for a targeted therapeutic approach with a wide range of pharmacological interventions, which, in the majority of cases, improves disease outcomes. Over the years, knowledge about the extra-articular manifestations of the disease has increased. Patients with RA may develop multiple extra-articular manifestations, particularly in cases where the disease is inadequately treated. The most common extra-articular manifestations are rheumatoid nodules (which, in turn, can be induced by treatment with methotrexate) and interstitial lung disease. However, patients are also more predisposed to the development of rarer conditions such as vasculitis and amyloidosis, which, in turn, can cause damage to the nervous structures [1].

Neuropathic involvement is increasingly recognized as belonging to the extra-articular manifestations of the disease. It is estimated that approximately 17–20% of RA patients (even in remission according to EULAR/ACR criteria) exhibit characteristics of neuropathic pain, scoring > 18 on the Pain Detect Questionnaire (PDQ) [2,3]. The presence of neuropathic-like pain features has been reported in the early stages of the disease and represents an unfavorable prognostic factor for achieving short-term remission [4].

The pathophysiological mechanisms underlying neuropathic symptoms are not yet fully understood. Peripheral pain mechanisms include the direct activation of nociceptors, as well as sensitization by joint inflammation. Local immune cells secrete cytokines and inflammatory mediators that act on the peripheral nerve terminals of nociceptors [5]. In response to inflammation mediators, intracellular signaling pathways lead to a cascade of phosphorylation, reducing the threshold for generating action potentials, and ultimately resulting in increased pain sensitivity. Various inflammatory cytokines, including tumor necrosis factor (TNF) alfa, interleukin (IL)-1β, IL-6, and IL-17, can directly alter nociceptor responses [6].

Among the extra-articular manifestations of rheumatoid arthritis, accelerated atherosclerosis must also be considered, the pathogenetic mechanisms of which largely overlap with those of the inflamed synovium [7]. The resultant endothelial damage can undoubtedly also promote peripheral nervous damage.

A portion of neuropathic symptoms may also be a direct consequence of synovial inflammation mechanisms affecting small nerve structures such as the digital nerves at the metacarpophalangeal joint level. Currently, high-resolution ultrasound systems equipped with very high-frequency probes allow for detailed visualization of the palmar digital nerves [8].

Based on these considerations, the objectives of this study were to evaluate, in patients with RA, the potential presence of dimensional alterations suggestive of neuropathy, particularly in terms of increased cross-sectional area (CSA) assessed by ultrasound, at the level of the palmar digital nerves, and to identify clinical, laboratory, and instrumental variables associated with an increased CSA of the palmar digital nerves.

## 2. Materials and Methods

### 2.1. Patients

We carried out a cross-sectional study at the Rheumatology Unit of the Polytechnic University of the Marche, “Carlo Urbani” Hospital in Jesi (Ancona). The center is a third-level reference for the diagnosis and treatment of RA. This study included consecutive adult patients diagnosed with RA in accordance with the ACR/EULAR 2010 criteria [9], regardless of disease activity status. Patients with coexisting conditions, which may be confounder variables for the ultrasound and clinical evaluations, were excluded, specifically those with connective tissue disorders, crystal-induced arthropathies (e.g., gout and calcium pyrophosphate crystal arthropathy), viral infections, poorly controlled endocrine disorders, and actively progressing neoplasms. Additionally, patients with conditions that could cause neuropathy were excluded from this study. This exclusion criteria encompassed individuals with coexisting carpal tunnel syndrome (CTS) or other entrapment neuropathies, neurologic diseases (such as multiple sclerosis, Parkinson’s disease or Alzheimer’s disease), vasculitis, type II diabetes mellitus, a history of alcoholism, and those using potentially neurotoxic medications.

### 2.2. Clinical, Laboratory, and Radiographic Assessments

At the time of inclusion in the study, the following clinical, laboratory, and radiographic data were recorded for each patient: age, gender, body mass index (BMI), duration of disease (years), tender joint count (TJC) (out of 28 joints), swollen joint count (SJC) (out of 28 joints), Patient Global Assessment of disease activity (PaGA) on an 11-point numerical scale, Visual Analogue Scale (VAS) for pain on an 11-point numerical scale, Physician Global Assessment of disease activity (PhGA) on an 11-point numerical scale, erythrocyte sedimentation rate (ESR) and C-reactive protein (CRP), serological assessment of rheumatoid factor (RF) and anti-citrullinated peptide antibodies (ACPA), presence/absence of radiographic erosive disease (evaluated in hands, wrists, and feet) according to the EULAR definition [10], comorbidities, and ongoing therapy. Clinical evaluation was conducted by a rheumatologist with over 10 years of experience in the diagnosis and treatment of RA (M.D.C.). The Clinical Disease Activity Index (CDAI) was calculated based on the collected clinical parameters, and disease activity status was identified. Patients were also assessed to estimate the functional capacity of the upper limb using the QuickDASH questionnaire, while the presence of neuropathic characteristics of painful symptoms was investigated using the PDQ.

The CDAI is a disease activity index derived from the algebraic sum of the following parameters: SJC (0–28) + TJC (0–28) + PaGA + PhGA. Due to its ease of calculation and not requiring laboratory parameters, the CDAI can be assessed at any time with a simple clinical evaluation of RA patients. The CDAI distinguishes four states of disease activity: remission, low disease activity, moderate disease activity, and high disease activity [11].

The Disability of the Arm, Shoulder, and Hand (DASH) scale is an important assessment tool based on a district questionnaire completed by the patient to evaluate upper limb function. To make this assessment shorter but still valid and reliable, the QuickDASH questionnaire was constructed, based on 11 questions instead of 30 (excluding the two optional modules). To complete the questionnaire, patients must refer to their ability to perform certain actions based on the previous week. The 11 items refer to specific actions performed with the upper limb, the interference it creates with the patient’s daily life, the experienced pain, and how much it compromises sleep function. All questions have the same response mode, based on a numerical scale ranging from 1 to 5 points, with increasing variability depending on the difficulty experienced for the required action (1 = ‘no difficulty’; 5 = ‘unable to do’). The final score, expressed in percentages (range of 0–100, higher scores correspond to greater disability), is the average score of the responses, subtracting 1, and multiplying by 25 [12]. 

The PDQ is a screening tool based on symptoms to identify neuropathic pain components in patients with chronic pain, also validated in RA. The questionnaire consists of seven questions addressing the quality of neuropathic pain symptoms; it is completed by the patient, and no physical examination is required. The first five questions assess the type of neuropathic symptoms present, and their severity is expressed with a score from 0 to 5 (never = 0, just noticed = 1, slightly = 2, moderately = 3, strong = 4, very strong = 5). Question 6 asks about the temporal course of pain, referring to visual patterns, and is assessed from −1 to 2, depending on the selected pain graph. Question 7 refers to a mannequin investigating pain radiation, with “yes” or “no” responses scoring 2 or 0, respectively. The total PDQ score ranges from a minimum of −1 to a maximum of 38 points. A score ≤ 12 indicates that the presence of a neuropathic pain component is unlikely (<15%), while values ≥ 19 indicate the presence of neuropathic pain as very likely (>90%). A score between these two values (13–18) represents uncertainty [13].

### 2.3. US Assessment

The ultrasound examinations were conducted blindly with respect to clinical evaluation by two operators with more than five years of experience in musculoskeletal ultrasound.

The ultrasound examinations were performed on the most clinically affected hand, as established by clinical assessment. The first rheumatologist (T.O.), blinded to clinical, laboratory, and radiographic data, assessed the ultrasound characteristics, predominantly those of an inflammatory nature, of the metacarpophalangeal joint from the second to the fifth finger.

The second operator (J.D.B.), blinded to the two previous evaluations (clinical and joint ultrasound), measured the cross-sectional area (CSA) of the palmar digital nerves (both nerves for each finger) at the base of the proximal phalanx from the second to the fifth finger of the hand investigated with joint ultrasound.

The images were obtained using a MyLab Class C ultrasound system (Esaote, Genoa, Italy), with a linear probe of 6–18 MHz for joint examination, and a “high-frequency” linear probe of 15–22 MHz for the examination of the palmar digital nerves.

#### 2.3.1. Acquisition of Articular Images

The metacarpophalangeal joints were examined in each patient with the hand resting on the examination table in a relaxed position, following the EULAR recommendations [14]. Longitudinal multiplanar dorsal scans were obtained, with adequate gel interposition to avoid compression. For each metacarpophalangeal joint, the presence/absence of synovitis was determined, both in grayscale, to assess the presence of synovial hypertrophy, and with the power Doppler (PD) technique. The PD settings were standardized (pulse repetition frequency 750 Hz; persistence 4; wall filter 3; Doppler frequency 9.1 MHz, gain set in order to not see color noise at the bony surface).

In the presence of synovitis, the grading of synovitis was determined in accordance with the definition of the EULAR Outcome Measures in Rheumatology Clinical Trials (OMERACT), with Grades 0–3: Grade 0, normal = absence of synovial hypertrophy regardless of the presence of effusion, absence of PD signal; Grade 1, mild synovitis = minimal synovial hypertrophy without protrusion from the line connecting the upper bony margins of the metacarpal head and the proximal phalanx, on PD examination up to three individual spots or one confluent spot and two individual spots or two confluent spots; Grade 2, moderate synovitis = synovial hypertrophy extending beyond the line but with a concave or flat superior surface, PD greater than Grade 1 but with signal involving less than 50% of the region with synovial hypertrophy; Grade 3, severe synovitis = marked synovial hypertrophy extending above the joint line and with a convex upper margin, PD signal involving more than 50% of the region with synovial hypertrophy [15] (Table 1). Finally, a composite US score for synovitis was obtained.

#### 2.3.2. Determination of Cross-Sectional Area (CSA) of Palmar Digital Nerves

**Figure 1:**
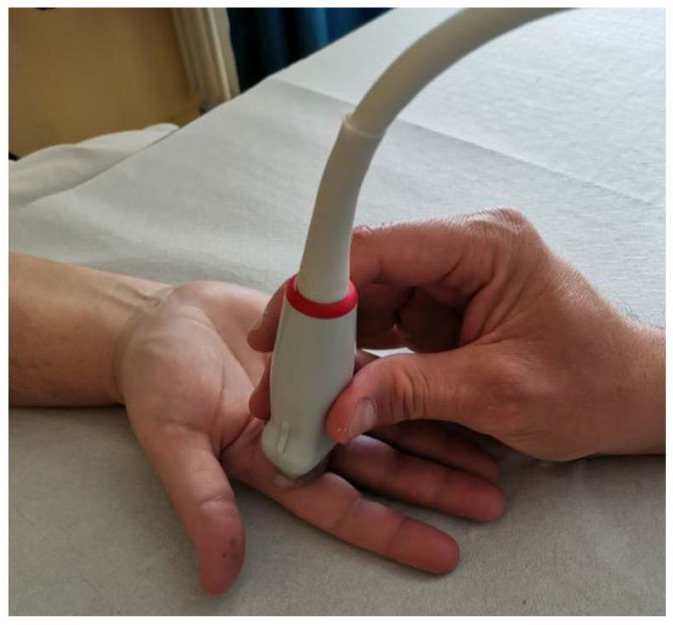
Scanning technique for the assessment of palmar digital nerves.

**Figure 2:**
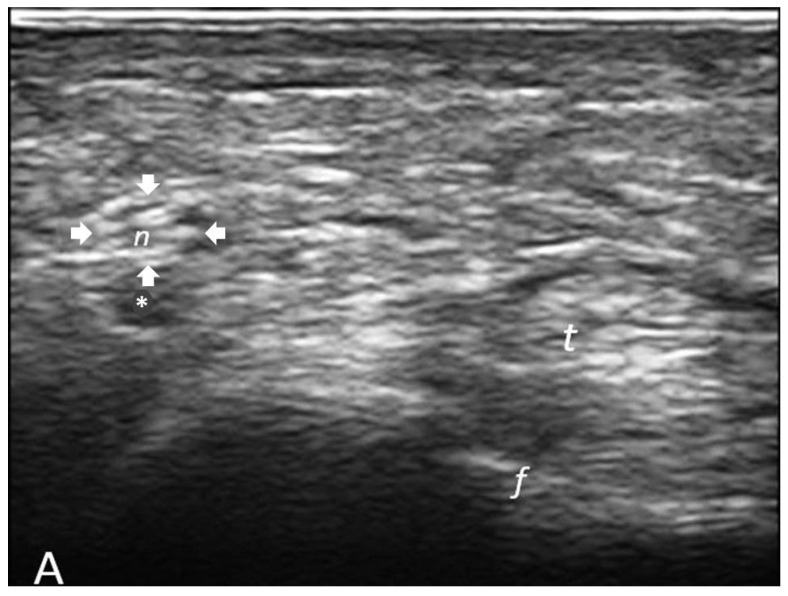

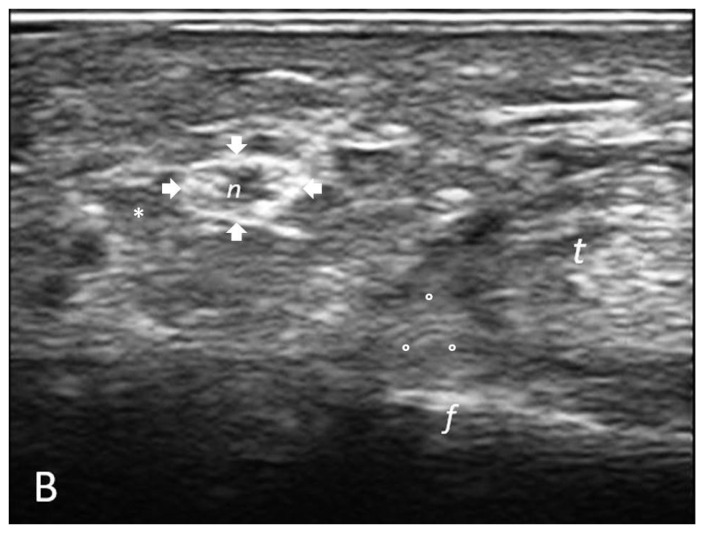
Ultrasound images of palmar digital nerves on the radial aspect of a patient with rheumatoid arthritis, right hand. In (**A**), the scan is performed at the level of the second finger with the metacarpophalangeal joint spared from the inflammatory joint process (ultrasound Grade 0). The cross-sectional area of the visualized palmar digital nerve is 1 mm^2^. In (**B**), the scan is conducted at the level of the third finger with the metacarpophalangeal joint affected by synovitis, as detected by ultrasound (ultrasound Grade 3). The CSA of the visualized nerve is 3 mm^2^. Legend: arrows outline the palmar digital nerves; circles (present only in (**B**)) identify hypertrophic synovial tissue at the metacarpophalangeal joint; t = flexor tendons of the fingers; f = base of the proximal phalanx; asterisk = palmar digital artery.

Both pairs of nerves (radial and ulnar branches) were studied for each joint. The direction of the ultrasound beam was maintained perpendicular to the nerve to avoid artifacts from anisotropy and deformation of the nerve itself. The ultrasound examination began by identifying anatomical landmarks such as the metacarpal head and the flexor tendons of the fingers (Figure 1). The palmar digital nerves run laterally to the flexor tendons of the fingers and appear as small structures adjacent to the palmar digital arteries (Figure 2).

The CSA (measured in mm^2^) of the palmar digital nerves was measured on axial images, at the base of the proximal phalanx using the trace function, determining a continuous line on the inner edge of the hyperechoic ring surrounding the nerve at the level of the corresponding metacarpophalangeal joint.

### 2.4. Statistical Analysis

Patient data were collected in an Excel database and analyzed using STATA 18. The data are presented as either mean and standard deviation (SD) or median and interquartile range (IQR), depending on the distribution. 

Statistical analysis was conducted by considering the sum of the CSAs of the eight palmar digital nerves (each pair of palmar digital nerves for each finger, from the second to the fifth finger) of the hand most clinically affected. Since there are no studies describing data about the CSA of palmar digital nerves in patients with RA, we estimated the required sample size considering a US study evaluating the CSA of the median nerve in patients with RA with and without carpal tunnel syndrome [16]. Assuming a mean sum of CSAs of palmar digital nerves in patients with RA, with and without active synovitis, of 13 and 9 mm^2^, respectively, a SD of 4 mm^2^ for both, and an alpha error of 5%, 44 patients would be required to observe a significant difference with a power of 90% or greater.

To investigate potential associations between the sum of the CSAs and other variables, analyses were conducted using Spearman’s rank correlation coefficient. Initially, the sum of the CSAs was compared with measures of disease activity (CDAI), neuropathic pain (PDQ), and functionality (QuickDASH). The sum of the CSAs was then compared with the ultrasound grading of synovitis.

Variables found to be correlated with the sum of the CSAs, considered as the dependent variable, were then included in multiple regression analyses. Since both the CDAI and the ultrasound grading were associated with the sum of the CSAs, and given the collinearity observed between CDAI and ultrasound grading of synovitis, two separate analyses were conducted. Among the independent variables, in addition to the already mentioned measures of disease activity, the duration of the disease, age, sex, BMI, presence of RF, presence of ACPA, presence of erosive disease, coexisting fibromyalgia, and coexisting osteoarthritis were considered. Statistical significance was considered for *p*-values less than 0.05.

## 3. Results

### 3.1. Sample Characteristics 

This study included 63 patients. The ultrasound examination was conducted on 252 metacarpophalangeal joints and on 504 palmar digital nerves, respectively.

Forty-seven (74.6%) patients were female, with a mean age (SD) of 64.1 (12.0) years, and a median disease duration (IQR) of 8.0 (6.0–16.0) years. 

The median (IQR) sum of CSAs of palmar digital nerves results were 17 (13–19) mm^2^, with a range from 8 to 31 mm^2^. Regarding disease activity, the median (IQR) CDAI score was 9.0 (4.0–18.0), and 29 (46.0%) patients demonstrated at least one metacarpophalangeal joint with an ultrasound score > 0. Erosive disease was detected in 60.3% of the patients. Radiographic evidence compatible with concomitant osteoarthritis of the metacarpophalangeal joints was observed in 27% of the patients, while 11% exhibited concurrent fibromyalgia. Table 2 summarizes the key characteristics of the study sample.

### 3.2. Correlation Analyses

**Figure 3:**
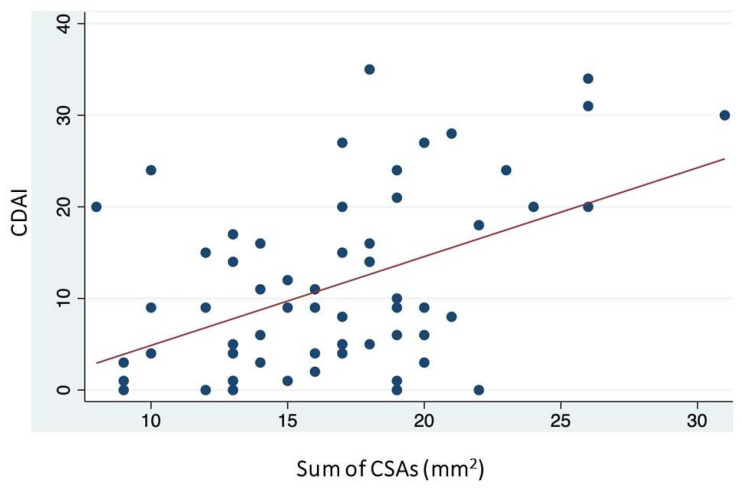
Interpolation line depicting the correlation between the Clinical Disease Activity Index (CDAI) scores and the sum of the cross-sectional areas (CSAs) of the palmar digital nerves.

In the correlation analyses between clinimetric indices and the sum of CSAs of palmar digital nerves, the CDAI was the only index to demonstrate a significant, albeit moderate, correlation (r = 0.387) (Figure 3). No significant correlations were found between the sum of CSAs and neuropathic pain, as assessed by PDQ, and with functional capacity, as evaluated by the QuickDASH (Table 3).

**Figure 4:**
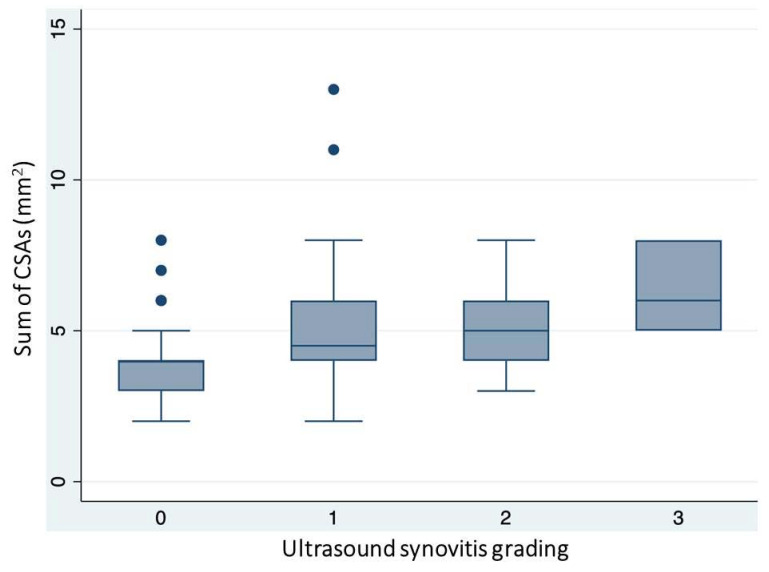
Box plots demonstrating the increase in median values of the sum of the cross-sectional areas (CSA) of palmar digital nerves with the increasing ultrasound grading of synovitis.

A second correlation analysis compared the sum of CSAs with synovitis scoring, considered for different grading. Here, a significant correlation was found between the sum of CSAs and the presence of synovitis across all evaluated gradings (1–3) (r = 0.381) (Figure 4), and to a lesser extent when considering only gradings 2 and 3 (r = 0.290). The same analysis also considered the CDAI to evaluate its correlation with ultrasound grading. The CDAI showed a moderate correlation (rho = 0.596) with the presence of ultrasound-detected synovitis (Grades 1–3) (Table 4).

### 3.3. Multivariate Analyses

In the multivariate analysis, using the sum of CSAs of palmar digital nerves as the dependent variable, the primary predictive variable identified was the CDAI (*p* < 0.001), followed by sex (*p* = 0.026), indicating a greater CSA of nerves in male patients (Table 5). No significant associations were found with age, BMI, duration of disease activity, serological or radiographic status, or the presence of nociplastic pain (fibromyalgia). Subsequently, a second analysis was conducted, in which the CDAI was replaced with the presence of ultrasound-detected synovitis (Grades 1–3). In this case, the presence of ultrasound-detected synovitis emerged as the main predictor of the sum of CSAs of palmar digital nerves, with sex also maintaining its significance (Table 6).

## 4. Discussion

To the best of our knowledge, this is the first study demonstrating morphological alterations in the palmar digital nerves, specifically the presence of increased CSA documented by ultrasound, in patients with “active” RA. These findings may, at least to some extent, explain the presence of neuropathic-like symptomatic features coexisting with nociceptive pain in RA patients with poorly controlled disease activity.

Modern imaging techniques, such as high-resolution ultrasound, enable the identification of anatomical details of joint and periarticular structures that were unimaginable until a few years ago [17].

Ultrasound is a technique that facilitates the real-time identification and highly precise localization of potential nerve damage. Presently, ultrasound research focused on nerves predominantly targets the structures of the upper limb. A lexical analysis of the literature has highlighted that the median nerve, followed by the ulnar nerve, are the two nerves upon which the majority of global research efforts are concentrated [18]. However, although the palmar digital nerves are infrequently mentioned in the international literature, their ultrasound examination can be meaningful, particularly in patients with RA, as demonstrated in this study.

In neurology, nerve ultrasound has demonstrated significant potential in the morphological study of nerves, especially the more superficial ones, and is increasingly being used as a complementary technique to electrophysiological studies. Inflammatory nerve pathologies typically increase the CSA of the involved nerve. Depending on the pathology, the increase in CSA can be focal, multifocal, regional, or diffuse [19].

In conditions of rheumatological interest, such as systemic sclerosis, ultrasound imaging also allows for the identification of morphological anomalies in nerves that are asymptomatic. A study from 2010, which utilized a linear probe capable of reaching up to 18 MHz, documented how the median nerve is increased in various dimensional parameters, including the CSA, in patients with systemic sclerosis compared to healthy controls. Considering that these patients did not present evident signs of tenosynovitis of the flexor tendons or synovitis of the radiocarpal joint, and only a small portion exhibited friction rubs, the pathogenetic mechanisms underlying the dimensional increase in the median nerve likely reside in ischemic events at the level of the vasa nervorum secondary to vasospasm [20].

The ultrasonographic probes currently available exceed 20 MHz and are capable of providing magnified images even of small structures that are poorly explored with lower frequencies. In this study, the use of a linear probe capable of reaching 22 MHz has documented alterations in the CSA of millimetric structures such as the palmar digital nerves. To date, the ultrasonographic study of the palmar digital nerves has been reserved for conditions such as the identification of traumatic or benign neoplastic lesions like schwannomas or neurofibromas [21]. However, extending ultrasound examination to palmar digital nerves may also play a role in chronic inflammatory joint conditions.

The ultrasound study of nerves in chronic articular inflammatory conditions is currently primarily focused on identifying local compression syndromes, such as CTS. Compressive conditions typically increase the CSA of the nerve by altering microvasculature, leading to Schwann cell necrosis, and subsequently demyelination under conditions of advanced damage [22].

A study comparing ultrasound characteristics of the median nerve in patients with RA versus those with idiopathic CTS revealed a significantly increased CSA of the median nerve in patients with idiopathic CTS. However, in patients with RA, inflammatory findings predominate, as expected, at the level of joint synovial structures and tendon sheaths, but also at the neural level, with the presence of intraneural PD signal [23].

In the absence of clear compressive mechanisms, inflammatory mechanisms of RA can probably affect neural structures. A 2008 study conducted on patients diagnosed with RA in accordance with the 1987 ACR criteria revealed the presence of electrophysiological signs of neuropathy in 57% of the patients. The majority of these cases exhibited axonal neuropathy, often in the absence of overt clinical manifestations. A subset of these patients underwent sural nerve biopsy, which, among other histopathological findings, showed perineural thickening, perivascular lymphomonocytic infiltrate, and loss of myelinated fibers. The clinical variables associated with neuropathy included the absence of tendon reflexes and the presence of other extra-articular manifestations. However, no associations were found between the presence of neuropathy and disease activity, disease duration, seropositivity, treatment, joint deformities, erosive damage, and joint deformities [24].

In the present study, the correlation between digital nerve CSA, CDAI, and ultrasound grading measures of disease activity, implies a certain association with the inflammatory burden. It can be hypothesized that cytokines, locally produced by inflamed synovial tissue in metacarpophalangeal joints, could impact adjacent structures such as the palmar digital nerves. For instance, it is known that injection of TNF alpha into nerves can induce Wallerian degeneration. Similarly, pro-inflammatory cytokines can lead to increased vascular permeability and alterations in the blood–nerve barrier [25].

The additional correlation that has emerged is with gender, specifically, a significantly higher nerve CSA was observed in men. Presumably, this correlation is not related to RA-specific characteristics in men but rather to anthropometric factors. Although there is a lack of data regarding palmar digital nerves, previous ultrasound studies conducted on healthy subjects have documented an increased CSA in men compared to women in different anatomical regions [26,27]. Theoretically, it was reasonable to consider a correlation between the PDQ and the CSA of nerves. However, the results of the study did not reveal such a correlation. It is likely that the PDQ, designed for identifying neuropathic pain in low back pain, is not the appropriate tool for assessing neuropathic pain originating from small-caliber structures such as the palmar digital nerves. Certain characteristics of neuropathic pain, such as radiation, are difficult to capture when the nerve alterations are at the digital level.

Additionally, high scores on the PDQ, indicative of probable neuropathic pain, can often be attributed to a concurrent presence of fibromyalgia [28].

Among the covariates considered for determining the CSA of nerves, fibromyalgia was also evaluated. However, its presence does not seem to be relevant in this context. Recent evidence suggests that in patients with fibromyalgia, the CSA of certain nerves (primarily the sural nerve and the vagus nerve) is increased. At the level of the palmar digital nerves, fibromyalgia does not appear to be capable of causing an increase in the CSA [29,30].

At present, the results of the study are not widely generalizable. The limitations primarily stem from the monocentric recruitment, with specific expertise in the use of ultrasound in managing patients with RA. These findings will need validation by other research groups utilizing high-frequency ultrasound probes. Furthermore, it will be necessary to assess the longitudinal changes of CSA to determine if disease activity control is associated with a reduction in CSA over time. Another limitation worth mentioning resides in the ultrasound examination, which relied solely on an assessment of the CSA of the palmar digital nerves based on transverse images, while longitudinal scans were not performed. However, longitudinal scans are hardly feasible for small structures (it is worth noting that, generally, in joints not affected by synovitis, the CSA of the palmar digital nerves is around 1 mm^2^) and would be excessively time-consuming even for experienced hands. Furthermore, the CSA assessed in transverse images remains the most reliable measure in detecting caliber alterations [31].

In future studies, it will also be interesting to verify whether, in addition to the proximity of the metacarpophalangeal joints, the CSA of the palmar digital nerves can be increased further distally, for example, at the level of the proximal interphalangeal joints, in the case of synovitis of the same. It will also be appropriate to assess whether, in the presence of synovitis in joint sites other than the metacarpophalangeal joints and in the absence of local compressive phenomena, not only the palmar digital nerves can be increased in terms of CSA, but also other nervous structures such as the radial or ulnar nerves, in the case of wrist synovitis, or the plantar digital nerves, in the case of synovitis of the metatarsophalangeal joints.

## 5. Conclusions

Extra-articular involvement in patients with RA is consistently considered, both to estimate the disease burden and to guide appropriate therapeutic choices. Nervous system involvement, to date, has been predominantly regarded in terms of entrapment neuropathies, with CTS being the most prominent, and to a lesser extent, as a consequence of systemic disease manifestations secondary to vasculitis. In this study, we aimed to demonstrate that inflammatory expressions of the disease can also have repercussions on neural structures. Thus, the synovitis present at the level of the metacarpophalangeal joints likely creates a local inflammatory environment capable of affecting the palmar digital nerves as well. This phenomenon occurs even in the absence of compressive mechanisms, such as the compressive neuropathy of the median nerve at a fibrous canal like the carpal tunnel.

In patients with RA, an increase in the CSA of the palmar digital nerves, detectable through ultrasound and indicative of potential neuropathic damage, is correlated with disease activity, measured both by composite disease activity indices (CDAI) and ultrasound grading of synovitis. A poorly controlled disease activity can create a perineural inflammatory environment with repercussions on the CSA of the palmar digital nerves. These aspects may, at least in part, explain the presence of neuropathic pain symptoms experienced by RA patients.

## Figures and Tables

**Table 1:** Synovitis grading according to the EULAR-OMERACT definition.

Synovitis Grade	Features
0	Normal joint: absence of synovial hypertrophy regardless of the presence of effusion, absence of power Doppler signal
1	Mild synovitis: synovial hypertrophy without protrusion from the line connecting the upper bony margins of the metacarpal head and the proximal phalanx, on power Doppler examination up to three individual spots or one confluent spot and two individual spots or two confluent spots
2	Moderate synovitis: synovial hypertrophy extending beyond the line but with a concave or flat superior surface, power Doppler greater than Grade 1 but with signal involving less than 50% of the region with synovial hypertrophy
3	Severe synovitis: marked synovial hypertrophy extending above the joint line and with a convex upper margin, power Doppler signal involving more than 50% of the region with synovial hypertrophy

**Table 2:** Sample characteristics.

Variables	Results
Age, years, mean (SD)	64.1 (12.0)
Sex, female, *n* (%)	47 (74.6)
Body mass index (kg/m^2^), mean (SD)	24.6 (4.5)
Coexisting osteoarthritis, *n* (%)	17 (27.0)
Coexisting fibromyalgia, *n* (%)	7 (11.1)
Disease duration, years, median (IQR)	8.0 (6.0–16.0)
RF presence, *n* (%)	48 (76.2)
ACPA presence, *n* (%)	49 (77.8)
Erosive disease, *n* (%)	38 (60.3)
CDAI, median (IQR)	9.0 (4.0–18.0)
Sum of CSAs, mm^2^, median (IQR)	17 (13–19)
Number of patients with at least one joint with ultrasound score > 0, *n* (%)	29 (46.0)
Number of patients with at least one tendon with ultrasound score > 0, *n* (%)	5 (7.9)
PDQ, median (IQR)	14.0 (9.0–20.0)
QuickDASH, median (IQR)	47.7 (34.1–65.9)
Steroid use, *n* (%)	24 (38.1)
- Low dose, *n* (%)	18 (28.6)
- High dose, *n* (%)	6 (9.5)
cDMARDs use, *n* (%)	43 (68.3)
- Leflunomide, *n* (%)	1 (1.6)
- Methotrexate, *n* (%)	34 (54.0)
- Hydroxychloroquine, *n* (%)	14 (22.2)
bDMARDs use, *n* (%)	43 (68.3)
- anti-tumor necrosis factor alpha, *n* (%)	20 (31.7)
- anti-interleukin 6, *n* (%)	7 (11.1)
- anti-CD20, *n* (%)	7 (11.1)
- anti-CTLA4, *n* (%)	14 (22.2)

Abbreviations. SD = standard deviation; RF = rheumatoid factor; ACPA = anti-citrullinated peptide antibodies; CDAI: Clinical Disease Activity Index; CSAs = cross-sectional areas; PDQ = PainDetect Questionnaire; IQR = interquartile range; cDMARDs = conventional disease-modifying anti-rheumatic drugs; bDMARDs = biologic disease-modifying anti-rheumatic drugs.

**Table 3:** Correlation analysis between total area of digital nerves and clinimetric indices (disease activity, neuropathic pain, functional capacity) (Spearman’s rho with underlying *p*-values).

	Area of Digital Nerves (Sum of CSAs)	CDAI	PDQ
CDAI	r = 0.387 *p* = 0.001		
PDQ	r = −0.049 *p* = 0.702	r = 0.312 *p* = 0.012	
QuickDASH	r = 0.128 *p* = 0.316	r = 0.585 *p* < 0.001	r = 0.560 *p* < 0.001

Abbreviations. CSAs = cross-sectional areas; CDAI = Clinical Disease Activity Index; PDQ = PainDetect Questionnaire.

**Table 4:** Correlation analysis between the total area of digital nerves and synovitis assessed through different ultrasound grading systems (Spearman’s rho with underlying *p*-values).

	Area of Digital Nerves (Sum of CSAs)	CDAI	Ultrasound Grades 1–3	Ultrasound Grades 2–3
CDAI	r = 0.387 *p* = 0.001			
Ultrasound Grades 1–3	r = 0.381 *p* = 0.002	r = 0.596 *p* < 0.001		
Ultrasound Grades 2–3	r = 0.290 *p* = 0.021	r = 0.308 *p* = 0.014	r = 0.596 *p* < 0.001	
Ultrasound Grade 3	r = 0.205 *p* = 0.106	r = 0.263 *p* = 0.037	r = 0.419 *p* < 0.001	r = 0.638 *p* < 0.001

Abbreviations. CSAs = cross-sectional areas; CDAI = Clinical Disease Activity Index.

**Table 5:** Multivariate analysis considering the sum of CSAs of palmar digital nerves as dependent variable and considering CDAI as index of disease activity.

Area of Digital Nerves (Sum of CSAs)	Coefficient	Standard Error	t	*p*	95% CI
CDAI	0.258	0.060	4.25	<0.001	0.136–0.381
Disease duration (years)	−0.070	0.071	−0.98	0.333	−0.214–0.074
Age	0.037	0.045	0.81	0.422	−0.054–0.128
Sex (male)	2.892	1.266	2.28	0.026	0.351–5.433
BMI	0.127	0.119	1.06	0.294	−0.113–0.367
ACPA presence	0.040	1.672	0.02	0.981	−3.315–3.395
RF presence	0.631	1.707	0.37	0.713	−2.793–4.056
Erosive disease presence	−0.350	1.381	−0.25	0.801	−3.121–2.421
Coexisting fibromyalgia	1.674	1.840	0.91	0.367	−2.017–5.367
Coexisting osteoarthritis	0.020	1.260	0.02	0.987	−2.510–2.550
constant	7.913	4.401	1.80	0.078	−0.917–16.745

Abbreviations. CSAs = cross-sectional areas; CI = confidence interval; CDAI = Clinical Disease Activity Index; BMI = body mass index; ACPA = anti-citrullinated peptide antibodies; RF = rheumatoid factor.

**Table 6:** Multivariate analysis considering the sum of CSAs of palmar digital nerves as dependent variable and considering ultrasound grading of synovitis as index of disease activity.

Area of Digital Nerves (Sum of CSAs)	Coefficient	Standard Error	t	*p*	95% CI
Ultrasound Grades 1–3	1.706	0.443	3.85	<0.001	0.817–2.596
Disease duration (years)	−0.107	0.075	−1.43	0.159	−0.259–0.043
Age	0.020	0.046	0.43	0.665	−0.073–0.114
Sex (male)	2.733	1.294	2.11	0.040	0.135–5.332
BMI	0.134	0.122	1.09	0.280	−0.112–0.380
ACPA presence	−2.633	1.779	−1.48	0.145	−6.203–0.936
RF presence	2.447	1.769	1.38	0.172	−1.101–5.997
Erosive disease presence	1.178	1.337	0.88	0.383	−1.506–3.862
Coexisting fibromyalgia	2.458	1.872	1.31	0.195	−1.299–6.216
Coexisting osteoarthritis	−0.541	1.289	−0.42	0.676	−3.128–2.045
constant	10.328	4.461	2.32	0.025	1.376–19.281

Abbreviations. CSAs = cross-sectional areas; CI = confidence interval; BMI = body mass index; ACPA = anti-citrullinated peptide antibodies; RF = rheumatoid factor.

## Data Availability

Study data are available upon reasonable request to the corresponding author.
